# No Financial Disincentive for Choosing More Healthful Entrées on Children’s Menus in Full-Service Restaurants

**DOI:** 10.5888/pcd10.120266

**Published:** 2013-06-06

**Authors:** Rebecca A. Krukowski, Delia West

**Affiliations:** Author Affiliation: Delia West, Fay W. Boozman College of Public Health, University of Arkansas for Medical Sciences, Little Rock, Arkansas.

## Abstract

Children are eating restaurant foods more than ever before, and price is among the top considerations for food choices. We categorized and enumerated entrées on children’s menus from 75 full-service restaurant chains to compare prices of more healthful and less healthful entrées to test the assumption that more healthful food is more expensive. The mean (standard deviation) price of more healthful entrées ($5.38 [$2.01]) was not significantly different from the price of less healthful entrées ($5.27 [$2.04]). In contrast to research demonstrating that more healthful foods tend to be more expensive in grocery stores, more healthful entrées on children’s menus in restaurants were not more expensive than less healthful entrées.

## Introduction

Children are eating foods away from home more frequently than ever before (1). Calories from full-service restaurants contribute 4% to American children’s total intake, and calories from fast-food restaurants contribute 8% to 11% (1). In addition, among adults, price is second only to taste as a consideration in making food choices (2). Although research has demonstrated higher prices for more healthful foods than for less healthful foods in grocery stores (3,4), it is not known whether more healthful entrées on restaurant children’s menus are more expensive than less healthful entrées.

## Methods

In this descriptive study, we used the Children’s Menu Assessment (CMA) (5) to categorize and enumerate more healthful and less healthful entrées on children’s menus gathered from full-service restaurants in the 200 top-grossing restaurant chains identified in 2009 by *Restaurants & Institutions* (6). We used Hoover’s (7) to identify restaurants classified as full-service (n = 90) according to code 722110 in the 2007 North American Industry Classification System (8). “Full-service” is defined as “establishments primarily engaged in providing food services to patrons who order and are served while seated (ie, waiter/waitress service) and pay after eating” (9). Each restaurant chain was contacted to determine whether a children’s menu was available, and if it was, a copy of the children’s menu was requested (by mail or e-mail) or picked up from the restaurant in the Little Rock, Arkansas, area, where the research team was located. To reduce the potential effect of price variations due to geography, we made every attempt to obtain a menu in the Little Rock area. If the children’s menu (including prices) was available only in print format and the nearest restaurant in the chain was located more than 50 miles from Little Rock, the children’s menu was obtained at a restaurant by a colleague nearby. We excluded 15 restaurant chains that were closed or not classified as full-service according to CMA classification or that did not have a children’s menu (Figure). Menus from the remaining 75 restaurant chains were collected in 2012, and entrée prices were recorded.

**Figure:**
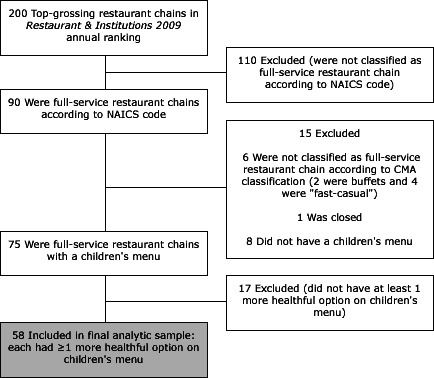
Inclusion and exclusion criteria for study on children’s menus. Abbreviations: NAICS, North American Industry Classification System; CMA, Children’s Menu Assessment.

CMA definitions of more healthful and less healthful entrées (Box) were based on guidelines established by the Nutrition Environment Measures Study in Restaurants assessment (10). Examples of “more healthful” entrées are pasta with marinara sauce; roasted, baked, or grilled chicken (alone or in a sandwich or salad); grilled salmon; and salad bars. Examples of “less healthful” entrées are hot dogs, hamburgers, macaroni and cheese, and fried chicken fingers. The CMA has high interrater and test–retest reliability (5).

**Box. Children’s Menu Assessment Guidelines for Determining Whether an Entrée is “More Healthful” (5)**

Guideline

Preparations such as “grilled,” “baked,” or “broiled” generally are considered a healthful choice (eg, grilled chicken), with the exception of grilled sandwiches (eg, grilled cheese).

Preparations such as “fried” are not considered healthful. Unless otherwise noted, fish and chicken entrées should be considered fried (eg, chicken fingers, chicken wings).

An item with cheese, butter, or cream sauce as a significant ingredient (eg, macaroni and cheese, cheese ravioli, pasta with butter) is generally not considered healthful.

An item with red meat is not considered healthful (eg, hamburger, taco, hotdog), unless it is specified that the meat is lower-fat or lower-calorie.

Only rate a sandwich as healthful if it is modified to be made with whole wheat bread, lower-calorie/-fat condiments (eg, light mayo) or all fruit preserves (lower sugar).

Only rate a daily entrée soup special as a healthful entrée if it is specified that the soup is consistent with the above guidelines for a healthful choice (ie, not made with cream, cheese, or red meat).

Green salads are considered a healthful entrée regardless of dressing, unless the protein source does not follow the other guidelines (eg, fried meat).

If the restaurant specifically promotes the salad bar for children (eg, a lower price for children to choose this option) and the price for this option is similar to other main dishes on the menu, count it as a healthful entrée salad, because the children are likely to have the option of choosing healthful components for their salad.

A vegetable plate can be counted as a healthful entrée salad if it is possible for the child to select all more healthful items for this plate (ie, more healthful items include nonfried vegetables, fruits without added sugar, low-fat dairy products, and whole grain items).

We enumerated the mean number of more healthful and less healthful entrées offered and the proportion of more healthful to less healthful entrées. Restaurants with the same price for all children’s entrées were identified. Salad entrées and nonsalad entrées were combined into 1 entrée category. Only children’s menus that had at least 1 more healthful and at least 1 less healthful entrée (n = 58) were included in comparisons of the number of more healthful and less healthful entrées and price comparisons of more healthful and less healthful entrées. We compared the number of more healthful entrées and mean price for the more healthful entrée(s) within each restaurant, respectively, to the number of less healthful entrées and mean price for the less healthful entrées by using paired sample *t* tests, with restaurant as the unit of analysis. One restaurant with a single more healthful entrée was excluded from the price comparison because the entrée price was “market price” (for fish). Data were analyzed using SPSS 20.0 (IBM, Chicago, Illinois).

## Results

All 75 restaurants had less healthful entrées on their children’s menu; 23% (n = 17) had only less healthful entrées (mean price, $4.89; standard deviation [SD], $1.09). Seventy-seven percent (n = 58) had at least 1 more healthful entrée and were thus included in the final analytic sample. On average, there were significantly fewer (*P* < .001) more healthful entrées (mean, 1.8; SD, 1.0) than less healthful entrées (mean, 8.2; SD, 3.9). The mean (SD) price of more healthful entrées ($5.38 [$2.01]) was not significantly different (*P* = .20) from the mean (SD) price of less healthful entrées ($5.27 [$2.04]). There was no price difference between more healthful and less healthful entrées for 14 restaurants because all children’s menu entrées were available for the same price. When excluding restaurants that had the same price for all children’s menu entrées, we found no significant difference (*P* = .20) between the mean (SD) price of more healthful entrées ($5.19 [$1.15]) and less healthful entrées ($5.05 [$1.19]).

## Discussion

Although most full-service restaurant chains in this sample had at least 1 more healthful entrée on their children’s menu (77%), we found few more healthful entrées on each children’s menu; thus, choice among more healthful entrées was limited. This is the first study to compare prices for making more healthful versus less healthful choices for entrées on children’s menus. We found no significant difference in mean price between more healthful and less healthful entrées on full-service restaurant children’s menus. However, it is still not known what other factors influence children’s menu choices or how a choice is made when an order is placed (eg, child vs parent decision).

This study had several limitations. The study did not examine prices for beverages, side dishes, or desserts; children’s menus frequently used a unit price for a combination of entrée, beverage, side dishes, and dessert and provided several options for side dishes or beverages at a constant price. Although the research team attempted to reduce the effect of potential price differences due to geographic location by using a standardized protocol for selecting proximal restaurant outlets, location-based price differences could have affected our results; other patterns may be found in other regions. In addition, this research focused on full-service restaurant chains that have a traditional handheld menu rather than a menu board (as fast-food and fast-casual restaurants do). It will be crucial in future research to examine whether prices differ between more healthful and less healthful entrées in fast-food and fast-casual restaurant chains, given their increasing caloric contribution to children’s diets (1).
